# Book Reviews

**Published:** 1874-03

**Authors:** 


### Book Reviews.

THE Student's Guide to Surgical Anatomy, being a Description of the most important Surgical Regions of the Human Body, and intended as an Introduction to Operative Surgery. By Edward Bellamy, F. R. C. S., Associate of King's College, London, &c. With illustrations. Pages 287. Philadelphia: Henry C. Lea, 1874.

The key-note of this valuable treatise is found in its introduction, where the student is reminded of "the necessity of making most careful inspection of the body, as a whole, before he attempts the more minute and detailed examination of its various parts. For this purpose, both the living model and the dead subject should be examined together. By the side of the body should be placed an entire articulated skeleton. Careful notice is to be taken of all the surface markings and of the superficial bearings of all prominent underlying structures."

It is a curious and remarkable fact, that the field of applied anatomy may be almost a *terra incognita* to him who has fully mastered the details of descriptive anatomy. This order of things was reversed in the day when a mistaken reverence for the human body proscribed its dissection, and when the plates, executed after nature by Andreas Vesalius, were unknown. Artists were the better anatomists of that period. Michael Angelo is said to have painted so boldly that he used his whole arm, rather than his fingers; and his drawings are anatomical studies to-day. Leonardo da Vinci sketched the pelvis at an angle of inclination to the vertebral column, which recent investigations only have proved to be correct.

We welcome Mr. Bellamy's work as a contribution to the study of regional anatomy, of equal value to the student and the surgeon. It is written in a clear and concise style; and its practical suggestions add largely to the interest attaching to its technical details. Of the fifty illustrations, some are taken from Fergusson, Heath, and Richet; but others are produced from drawings on wood by the author, and are certainly equal to the others in point of excellence and originality. Such, for example, as "the vertical section of the thorax through the clavicle, showing the relations of the subclavian vessels," and "the diagrammatic section of the shoulder-joint," give us an appreciation of the privileges of the students who attend the lectures on Operative Surgery at Charing Cross Hospital. After an inspection of these plates, the anatomist cannot avoid the conclusion that he has seen them before, in the cadaver; and cannot but wonder that they have hitherto failed of representation in the various treatises on surgical anatomy.

We cannot avoid quoting the concluding phraseology of one paragraph, as it gives expression to a thought which has occurred to the minds of many who have returned to their old dissecting-rooms with a riper experience than in the day when they assiduously labored there:

"It is often the end and aim of the dissector to make a clean or 'pretty' preparation, in following out the various vessels, nerves, etc., and for this purpose it is quite right that all pains be taken; but the student must remember that the more he cleans, the more he destroys the actual relation of the parts as they would be met with in an operation; and, moreover, he must remember that the very fasciæ he so studiously removes, are of the very greatest importance in surgical anatomy; and their removal destroys surgical continuity."

The volume is well printed, and will doubtless re-appear in several editions. J. N. H.

---

A Practical Treatise on the Diseases of Children. By J. Forsyth Meigs, M.D., and William Pepper, M.D. Fifth edition, revised and enlarged. Philadelphia: Lindsay & Blakiston, 1874.

This work is undoubtedly familiar to all of our readers. In its previous editions it has been fully reviewed in THE EXAMINER.

The chapters on the treatment of scarlet fever, and on variola and the vaccine diseases, have been re-written, and the views expressed somewhat modified from the previous editions. Articles on pulmonary emphysema, pneumothorax, affections of the tonsils, retro-pharyngeal abscess, malarial fever, and scrofula, have also been added. This increases the size of the volume to a little over 1,000 pages.

---

A Dictionary of Medical Science; with the Accentuation and Etymology of the Terms, and the French and other Synonyms. By Robley Dunglison, M.D., LL.D. A New Edition, enlarged and thoroughly revised by Richard J. Dunglison, M.D. Philadelphia: H. C. Lea, 1874.

For forty years this work has maintained its position as the standard medical lexicon of the English language. The author was engaged in the revision of the work, preparatory for the present edition, when prostrated by the prolonged illness which terminated his life. The work was continued by his son, Richard J. Dunglison; and by him the revision and amplification have been thoroughly and conscientiously carried out.

The present edition contains a large amount of new matter, including more than six thousand subjects and terms not treated in the previous editions, and increasing the size of the volume about one hundred and fifty pages.

It is a volume that forms a necessary part of every physician's library.

---

FOR SALE CHEAP.—A Physician's residence and practice in a country village.

Address S. H. DRAKE, M.D.,
*Rossville, Allamakee Co., Iowa.*

---

A RARE CHANCE FOR A GOOD PHYSICIAN.—I wish to dispose of my house and lot, in the town of Grayville, White county, Ill., together with as good a country ride as "Egypt" affords. Said house, lot, and barn, is pleasantly located, with good well and cistern, one block from railroad depot, and one door from the best hotel in town. For particulars, call on Drs. W. H. Byford, or Davis, Chicago, or address

J. MILLIRON, M.D.,
*Grayville, Ill.*

